# Heat Treatment’s Vital Role: Elevating Orthodontic Mini-Implants for Superior Performance and Longevity—Pilot Study

**DOI:** 10.3390/dj12040103

**Published:** 2024-04-11

**Authors:** Tinela Panaite, Carmen Savin, Nicolae Daniel Olteanu, Nikolaos Karvelas, Cristian Romanec, Raluca-Maria Vieriu, Carina Balcos, Madalina Simona Baltatu, Marcelin Benchea, Dragos Achitei, Irina Zetu

**Affiliations:** 1 Department of Oral and Maxillofacial Surgery, Faculty of Dental Medicine, “Gr. T. Popa” University of Medicine and Pharmacy, 16 Universitatii Str., 700115 Iasi, Romania; tinela-panaite@umfiasi.ro (T.P.); daniel.olteanu@umfiasi.ro (N.D.O.); karvelas93@gmail.com (N.K.); raluca-maria.vieriu@umfiasi.ro (R.-M.V.); carina.balcos@umfiasi.ro (C.B.); irina.zetu@umfiasi.ro (I.Z.); 2 Faculty of Materials Science and Engineering, “Gheorghe Asachi” Technical University of Iasi, 41 “D. Mangeron” Street, 700050 Iasi, Romania; madalina-simona.baltatu@academic.tuiasi.ro; 3 Faculty of Mechanical Engineering, “Gheorghe Asachi” Technical University of Iasi, Blvd. Dimitrie Mangeron, No. 61–63, 700050 Iasi, Romania; marcelin.benchea@tuiasi.com; 4 Department of Technologies and Equipments for Materials Processing, Faculty of Materials Science and Engineering, Gheorghe Asachi Technical University of Iaşi, Blvd. Mangeron, No. 51, 700050 Iasi, Romania; dragos-cristian.achitei@academic.tuiasi.ro

**Keywords:** dental mini implants, elastic modulus, heat treatment, stress shielding, stainless steel 316L, Ti6Al4V

## Abstract

Orthodontic mini-implants are devices used for anchorage in various orthodontic treatments. We conducted a pilot study which aimed to observe preliminary trends regarding the impact of heat treatment on the elastic modulus of Ti6Al4V alloy and stainless steel 316L mini-implants. The initial phase involved testing the impact of heat treatment on the mechanical properties of Ti6Al4V alloy and stainless steel 316L mini-implants. Material and methods: Ten self-drilling mini-implants sourced from two distinct manufacturers (Jeil Medical Corporation^®^ and Leone^®^) with dimensions of 2.0 mm diameter and 10 mm length were tested. They were separated into two material groups: Ti6Al4V and 316L. Using the CETRUMT-2 microtribometer equipment, indentation testing was conducted employing a diamond-tipped Rockwell penetrator at a constant force of 4.5 N. Results: Slight differences were observed in the elastic modulus of the Ti6Al4V alloy (103.99 GPa) and stainless steel 316L (203.20 GPa) compared to natural bone. The higher elastic moduli of these materials indicate that they are stiffer, which could potentially lead to stress-shielding phenomena and bone resorption. Heat treatment resulted in significant changes in mechanical properties, including elastic modulus reductions of approximately 26.14% for Ti6Al4V and 24.82% for 316L, impacting their performance in orthodontic applications. Conclusion: Understanding the effects of heat treatment on these alloys is crucial for optimizing their biomechanical compatibility and longevity in orthodontic treatment. To fully evaluate the effects of heat treatment on mini-implants and to refine their design and efficacy in clinical practice, further research is needed.

## 1. Introduction

Mini-implants are widely used in orthodontic treatments for providing anchorage. The mechanical characteristics of mini-implants play a crucial role in their success. The primary stability of mini-implants is essential for their successful integration with bone tissue. Factors such as bone physiology, implant size, shape, and surface characteristics influence the stability of mini-implants [1]. Mini-implant stability is crucial, and issues like managing stability and fracture resistance need to be addressed [2]. Microindentation is a valuable technique for assessing the durability and mechanical properties of various materials. This technique is crucial for determining mechanical properties at different scales, aiding in material design and assessing durability against degradation [3].

The incorporation of mini-implants into orthodontic treatment planning strategies has streamlined early anchorage control and enhanced effectiveness in addressing significant disparities in both skeletal and dental dimensions [4]. The survival rate of mini-implants is statistically influenced by the insertion torque, with higher values of torque potentially leading to stress, necrosis, and local ischemia [5]. 

Depending on the type of mini-implant and the driver shaft used, various fracture patterns were observed: nearly all types of mini-implants fractured around the acrylic block in the threaded part region of the mini-implants [6]. Herrera and Diez-Perez [7] have emphasized the significance of microindentation in the analysis of bones, showcasing its capability to augment current techniques and offer insights into the strength of materials when subjected to pressure. Furthermore, microindentation has been employed to examine the creep characteristics of materials, indicating its relevance in determining the long-term performance of materials under stress. This reinforces the concept of applying microindentation to assess the durability of orthodontic mini-implants against focused loads [8].

The importance of using microindentation is emphasized in its effectiveness in quantifying material stiffness, which is vital for evaluating its capacity to withstand various loads [9], thereby highlighting the potential of microindentation as a valuable tool for evaluating the mechanical properties of materials used in orthodontic mini-implants. This method enables the evaluation of hardness and elastic modulus, offering insights into the material’s ability to resist deformation and withstand mechanical stresses [10].

Microindentation is a technique that has been used since the 1950s, and in recent years, micro- and nanoindentation methods have been developed. Research focus has shifted towards the development of compatible mini-implants, which exhibit peri-implant biomechanics with a modulus of elasticity close to that of bone tissue and compression strength, while promoting appropriate osteoimmunomodulatory responses [11]. The Young’s modulus of titanium alloy is much higher than that of the surrounding bone tissue. The difference in Young’s modulus provides protection against tension when a load is applied. For example, the Young’s modulus of Ti6Al4V alloy is approximately 110 GPa, much higher than that of natural bone tissue—the Young’s modulus of cortical and trabecular bone ranges from 0.5 to 20 GPa [12]. The optimal stress for bone tissue generally falls between 20 and 60 MPa. If the stress level exceeds this range, bone tissue will undergo damage and subsequently be absorbed. Protecting against stress leads to unused atrophy of the affected bone tissue, and the implant tends to fail [13]. Therefore, reducing the Young’s modulus of the mini-implant is necessary to transfer stress to the surrounding bone tissue and ensure the long-term stability of the implant.

Analyzing the influence of Young’s modulus modification on orthodontic mini-implants made of Ti6Al4V and 316L alloys, through the examination of mechanical properties and the interpretation of results obtained using the microindentation technique using CETR UMT-2 microtribometer equipment, will significantly contribute to a more detailed understanding of the behavior of these materials and to identifying critical aspects related to resistance to concentrated loads. Hence, the aim of this research was to conduct a thorough examination of the mechanical characteristics of orthodontic mini-implants crafted from Ti6Al4V and 316L alloys post heat treatment via a UTTIS electric oven, employing established procedures for microindentation testing. The indentation process utilized a diamond-tipped Rockwell penetrator at a consistent force of 4.5 Newton. In this pilot study, we aimed to observe preliminary trends regarding the impact of heat treatment on the elastic modulus of Ti6Al4V alloy and stainless steel 316L mini-implants.

## 2. Materials and Methods

### 2.1. Study Design and Setting

In this study, we used 10 self-drilling mini-implants from two different brands of manufacturing companies (Jeil Medical Corporation^®^ Seul, Korea, and Leon^®^ Firenze, Italy) with diameters of 2.0 mm and lengths of 10 mm. The mini-implants were made of a titanium alloy, Ti6Al4V (titanium grad V), and stainless steel, 316L. These mini-implants were divided into two groups based on material: Ti6Al4V and 316 L. This pilot study investigated the impact of heat treatment on the mechanical properties of Ti6Al4V alloy and stainless steel 316L mini-implants.

**Figure 1:**
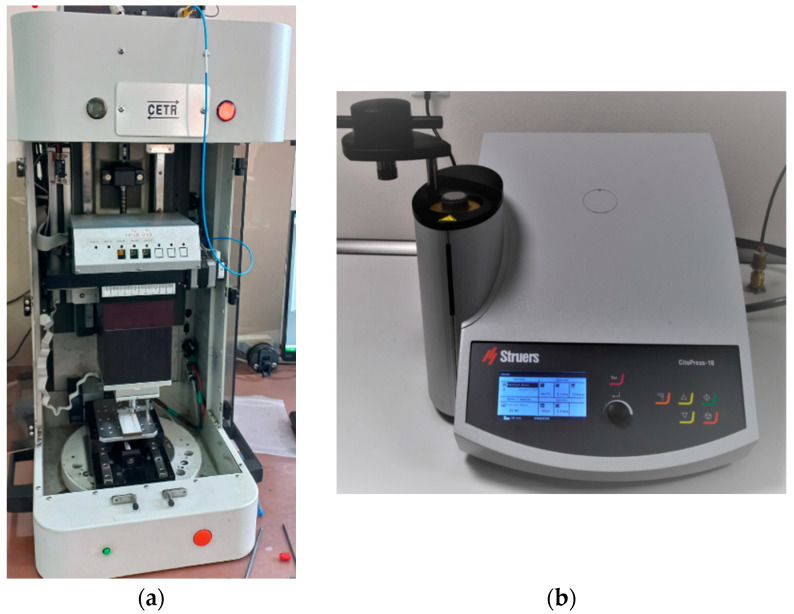
Equipment used for sample analysis: (**a**) CETR-UMT-2 microtribometer and (**b**) CitoPress equipment from Struers.

**Figure 2:**
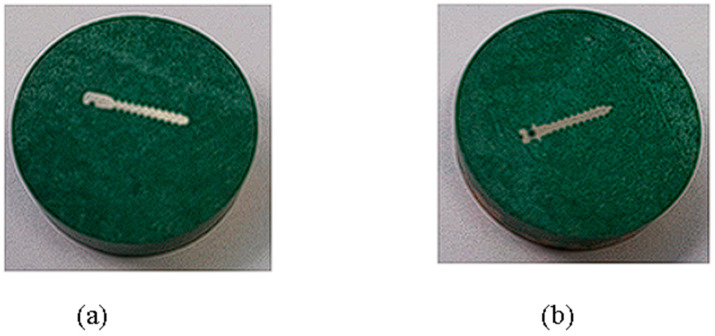
Samples of mini-implants hot-embedded and prepared for investigation through the indentation method (**a**) 316L and (**b**)Ti6Al4V alloy.

To conduct the microindentation test, we used the CETR UMT-2^®^ microtribometer equipment (CETR—Centre for Tribology Inc., Campbell, CA, USA) (Figure 1a), available in the Tribology Laboratory, Faculty of Mechanical Engineering, “Gheorghe Asachi” Technical University of Iasi, Romania. To efficiently embed samples with orthodontic mini-implants, hot-embedding followed by preparation for investigation through the microindentation method was chosen (Figure 2a,b). Initially, the samples were hot-embedded using the CitoPress^®^ equipment from Struers (Struers Company, Ballerup, Denmark) (Figure 1b). In this process, the samples were incorporated into a special resin, PuriFast, also purchased from Struers, because this method ensures the uniform and solid embedding of the samples, providing a stable base for subsequent processing stages, like the analysis of the samples through the indentation method. Embedding ensures that the samples are stable and well supported during analysis, which is important for obtaining precise results. After the samples were embedded, they underwent a grinding process until a mirror-like finish was achieved using the Tegramin equipment from Struers. Through fine grinding, the sample surface became extremely smooth and prepared for detailed analysis.

### 2.2. Mechanical Procedure

**Figure 3:**
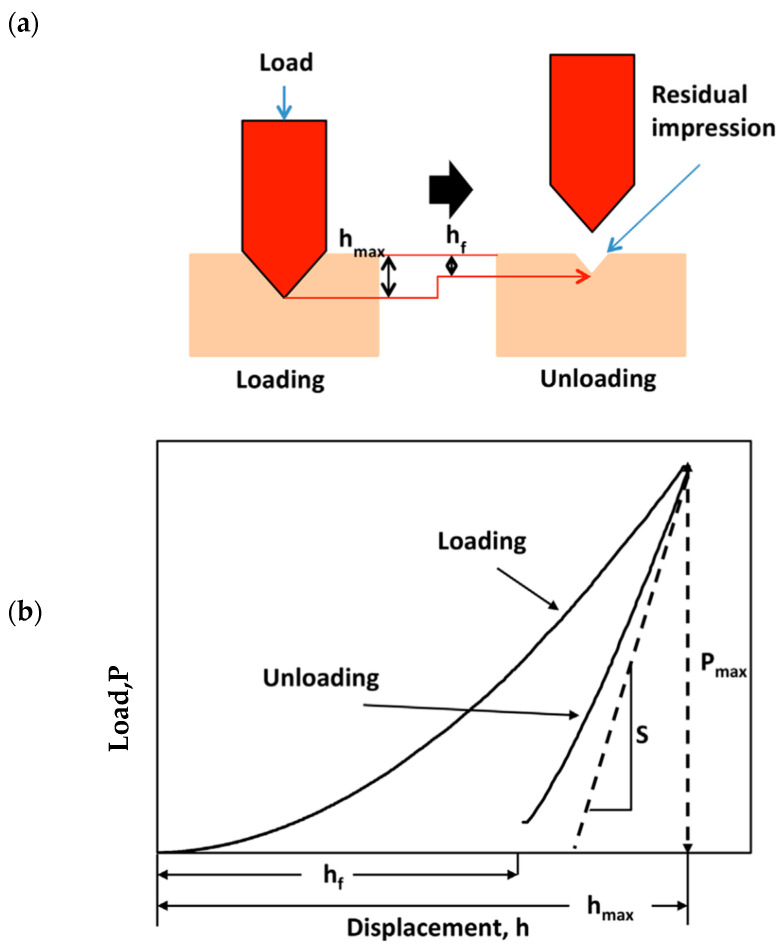
The measurement principle of the microindentation test: (**a**) Diagram illustrating working principles, (**b**) load–displacement curve showing the loading and unloading process, where S is the unloading contact stiffness. Figure 3 is reproduced with permission from Zhifeng Ren; Phys. Status Solidi A, published by Wiley Online Library, 2015 [14].

The microindentation method was used to determine the longitudinal elastic modulus of the Ti6Al4V alloy and 316L mini-implants. This method involves the penetration of the specimen’s surface with a conical-tipped indenter under a specific force. During the microindentation test, the values of the applied forces are recorded as a function of the depth of penetration of the indenter into the material. The measurement principle of the microindentation test is illustrated in Figure 3a,b.

To perform the microindentation test on the tribometer, a linear stage was installed with the sample fixed onto it. A capacitive sensor support was attached to this stage for recording the depth of penetration into the test material. A force sensor was used, and the rigid support in which the system was mounted was secured with two screws.

### 2.3. Statistical Analysis

The collected data were inputted into a Microsoft Excel 365 spreadsheet and subsequently imported into SPSS software (Version 28, IBM Corp., Armonk, NY, USA) for statistical analysis. The analysis process involved the following steps: (a) Initially establishing a database for descriptive statistics. (b) Employing qualitative variable analysis methods such as the Wilcoxon Signed-Rank Test. The reason for this was that the Wilcoxon Signed-Rank test is a non-parametric test used to compare paired samples when the assumption of normality is violated or when the data are ordinal. In this study, we compared the mechanical properties of Ti6Al4V and 316L mini-implants before and after heat treatment. It was applicable because it assessed whether there were significant differences in the mechanical properties of the Ti6Al4V and 316L mini-implants before and after heat treatment separately. The statistical significance threshold for establishing a relationship as significant was set at a probability value of *p* < 0.05.

### 2.4. The Experimental Method Involves the Following Steps

(a) Bringing an indenter with precisely defined geometry into contact with the sample under a known load and measuring the depth at which the indenter penetrates the sample;
(b) Reducing the load after reaching a maximum value, followed by monitoring the withdrawal of the indenter;
(c) Determining the elastic modulus of the sample by analyzing the load–unload curves and knowing the geometry of the indenter.

**Figure 4:**
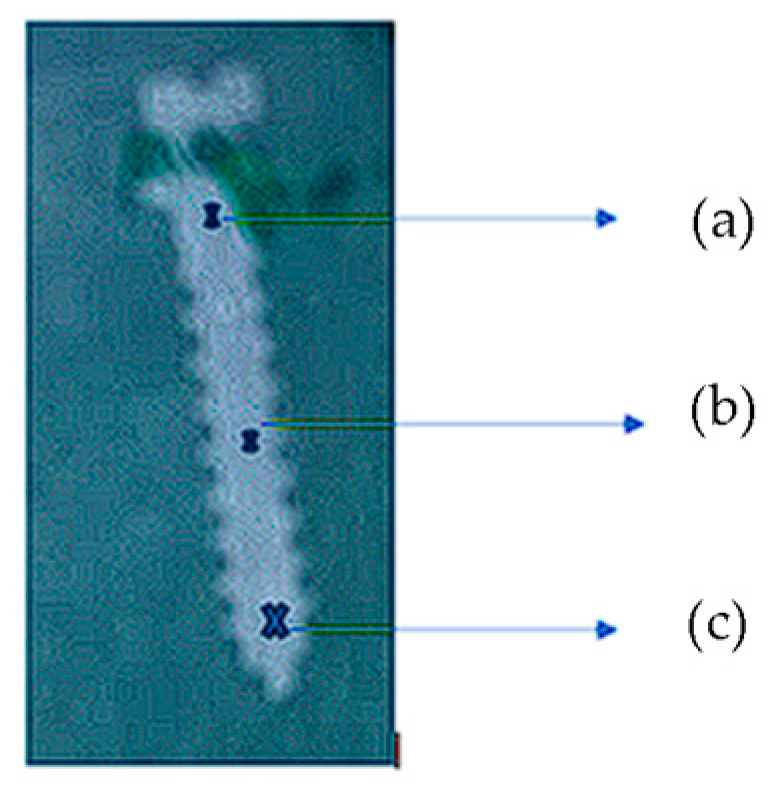
Indentation pressure zones: (**a**) the cervical region at the neck level; (**b**) the second one in the middle of the mini-implant; and (**c**) at the tip of the mini-implant.

These investigated samples were secured onto a flat surface of the testing device with screws and clamping clips. The tests were conducted under dry conditions, and for indentation, a diamond-tipped Rockwell-type penetrator with a cone angle of 120° and a spherical tip with a radius of 200 μm was used, applying a force of 4. 5 N. To ensure the most accurate determination, three distinct determinations were performed for each sample along the entire length of the mini-implant. Three measurement points were selected for analysis in the following areas of the orthodontic mini-implant: the cervical region at the neck level, the second one in the middle of the mini-implant, and at the tip of the mini-implant (marked with X in Figure 4a–c).

Upon completion of the work stages and their recording by the UMT 2 apparatus software, the indentation curves (depth vs. force) of the Ti6Al4V and 316L mini-implant samples were plotted using the UMT VIEWER TEST^®^ program (CETR—Centre for Tribology Inc., Campbell, CA, USA).

### 2.5. Modification of Properties through Heat Treatment

**Figure 5:**
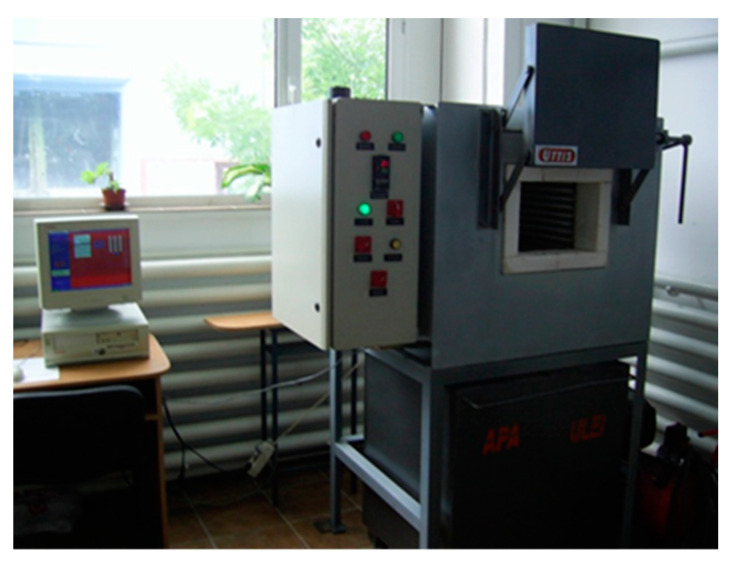
UTTIS electric oven.

We applied heat treatments to the experimental samples using the UTTIS-type electric oven (Figure 5), aiming to observe how the properties of the materials changed.

**Figure 6:**
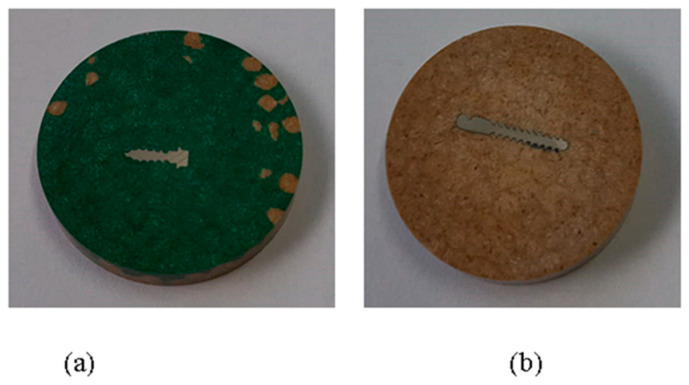
Samples of mini-implants after heat treatment for investigation through the indentation method: (**a**) Ti6Al4V and (**b**) 316L alloy.

For heat treatments, mini-implants purchased from, Dual Top Anchor System^®^ (Jeil Medical Corporation Seongnam, Seoul, Republic of Korea), and Leone^®^ (Leone S.p.A, Florence, Italy) were used (Figure 6a,b).

### 2.6. The Working Parameters Included

(1) Heating the electric oven (Figure 5) to 890 °C;
(2) Maintaining constant heat for 120 min;
(3) Slow cooling of the mini implants;
(4) After the heat treatments, the mini-implants were hot-embedded (Figure 6a,b) using the CitoPress equipment from Struers (Figure 1b).

### 2.7. Advantages of the Working Method Include

(1) High resolution: Microtribometers are capable of measuring at the nanometer scale, allowing for the precise characterization of the mechanical properties of materials on a small scale.
(2) Versatile samples: This method can be applied to a wide variety of materials, including polymers, metals, ceramics, and composites.
(3) Non-invasive: The indentation procedure is usually non-invasive, ensuring that the sample is not significantly destroyed or altered.
(4) Speed: Tests can be performed in a relatively short period compared to other testing methods.
(5) Flexibility: The elastic modulus, hardness, and other properties can be conveniently obtained through a single measurement.

## 3. Results

### Sample Processing Results

**Figure 7:**
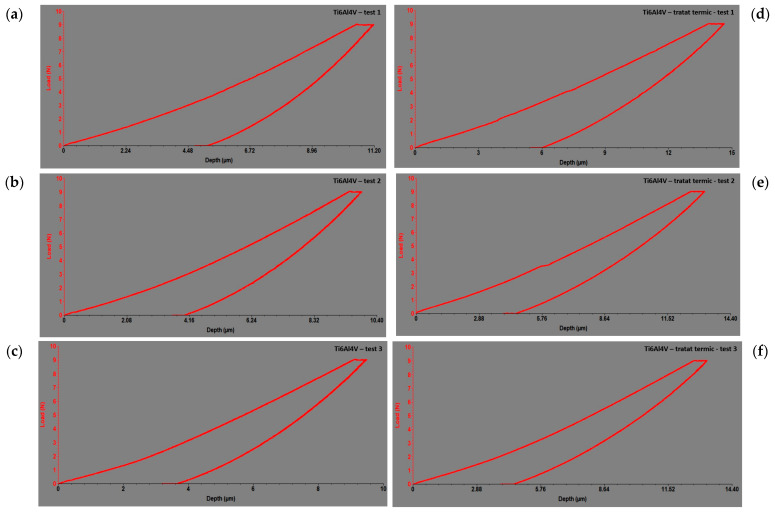
The force–depth curves of microindentation tests for the investigated Ti6Al4V alloy: (**a**) before heat treatment in the cervical region at the neck level; (**b**) before heat treatment in the middle of the mini-implant; (**c**) before heat treatment at the tip of mini-implant; (**d**) after heat treatment in the cervical region at the neck level; (**e**) after heat treatment in the middle of the mini-implant; (**f**) after heat treatment at the tip of mini-implant.

The results of the indentation test before heat treatment are presented in Table 1, and the resulting curves for Ti6Al4V (Figure 7a) can be found in Figure 7b–d. The modulus of elasticity and hardness are calculated by summing the three obtained values.

Descriptive statistics provided (Table 1): (1) The maximum load difference for the Ti6Al4V sample was 0.01 N, indicating a minor change in load capacity after treatment. (2) Before treatment, the maximum displacement for the Ti6Al4V sample was 8.22 µm, which increased to 9.21 µm after treatment, resulting in a difference of 0.99 µm. (3) The modulus of elasticity before treatment for the Ti6Al4V sample was 105 GPa, which decreased to 76.81 GPa after treatment, showing a reduction of 28.19 GPa. (4) Similarly, for the 316L sample, the maximum load before treatment was 4.50 N, which decreased slightly to 4.49 N after treatment. (5) The maximum load difference for the 316L sample was −0.01 N, indicating a slight decrease in load capacity after treatment. (6) Before treatment, the maximum displacement for the 316L sample was 6.62 µm, which increased to 7.54 µm after treatment, resulting in a difference of 0.92 µm. (7) The modulus of elasticity before treatment for the 316L sample was 210 GPa, which decreased to 157.87 GPa after treatment, showing a reduction of 52.13 GPa. These changes in mechanical properties highlight the impact of the heat treatment process on both the Ti6Al4V and 316L samples, with notable differences observed in the load capacity, displacement, and modulus of elasticity. The Wilcoxon test results show the test statistics and associated *p*-values for the Ti6Al4V alloy and 316L mini-implants, indicating a significant difference in the mechanical properties of both materials before and after heat treatment. The low values of the test statistic and the *p*-values (0.0185 in both cases) suggest that the observed difference between the data groups is unlikely to be due to random variability and is more likely to be statistically significant. The results obtained from the indentation test conducted on the Ti6Al4V titanium alloy and the 316L stainless steel offer valuable insights into their suitability for orthodontic mini-implants. The Ti6Al4V alloy had an elastic modulus of 105 GPa, while the 316L stainless steel had a modulus of 210 Gpa (Table 1). The elastic modulus is an important indicator of material stiffness, with a higher value indicating greater rigidity. In this comparison, 316L exhibited greater stiffness than Ti6Al4V. Although the difference was minor, it could impact the behavior of implants within the oral cavity.

There was a significant decrease in hardness from 0.87 GPa to 0.63 GPa, indicating lower resistance to plastic deformation. In Figure 7a–c, results of the alloys’ response during indentation tests before heat treatment are shown, and in Figure 7d–f, images after heat treatment are presented, in the form of force–displacement dependencies for Ti6Al4V. These dependencies are graphically expressed by indentation curves, illustrating how the applied force varies with the depth of the indenter penetration.

In Figure 7a–f, the responses of the alloys during the indentation tests are depicted as force–depth dependencies. These graphs provide a detailed perspective on how the applied force varies with the depth of penetration of the indenter into the tested material for each alloy individually. By analyzing these dependencies, a deeper understanding of the material’s behavior under indentation loads and its mechanical properties can be gained. Thus, these data provide essential information for evaluating and comparing the performances of different alloys regarding deformation resistance and stiffness.

**Figure 8:**
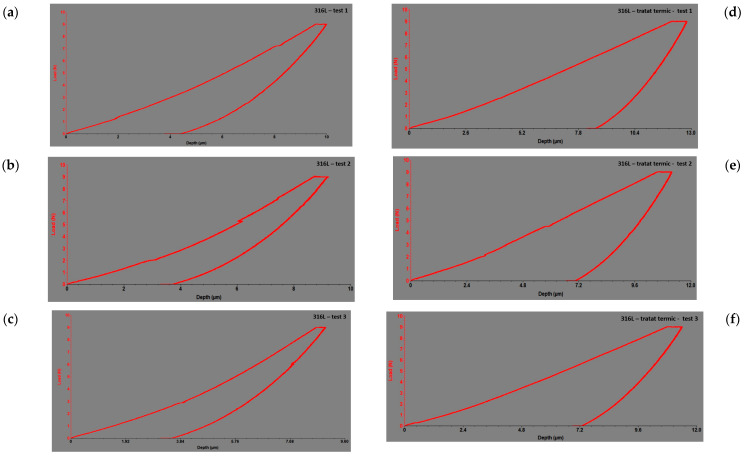
The force–depth curves of microindentation tests for the investigated 316L alloy: (**a**) before heat treatment in the cervical region at the neck level; (**b**) before heat treatment in the middle of the mini-implant; (**c**) before heat treatment at the tip of the mini-implant; (**d**) after heat treatment in the cervical region at the neck level; (**e**) after heat treatment in the middle of the mini-implant; (**f**) after heat treatment at the tip of the mini-implant.

Figure 8 presents images of the results of the alloy response during indentation tests before (Figure 8a–c) and after heat treatment (Figure 8d–f), in the form of force–depth dependencies for 316L.

In Figure 7a–f and Figure 8a–f, the responses of the alloys during the indentation tests are depicted as force–depth dependencies. These graphs provide a detailed perspective of how the applied force varied with the depth of penetration of the indenter into the tested material for each alloy individually. By analyzing these dependencies, a deeper understanding of the material’s behavior under indentation loads and its mechanical properties can be gained. Thus, these data provide essential information for evaluating and comparing the performances of different alloys regarding deformation resistance and stiffness.

## 4. Discussion

The aim of this pilot study was achieved through an examination of mechanical properties and the interpretation of the obtained results by using the microindentation technique with CETR UMT-2 microtribometer equipment. The study is expected to contribute significantly to a more detailed understanding of the behavior of these materials and to identify critical aspects related to resistance to concentrated loads. This suggests that the heat treatment had a notable impact on the mechanical characteristics of the mini-implants for both materials.

Descriptive statistics indicate notable changes in the mechanical properties of both the Ti6Al4V and 316L mini-implants after heat treatment, reflecting the outcomes of this pilot study. Ti6Al4V showed a slight increase in maximum load but a significant decrease in the modulus of elasticity, whereas 316L displayed a slight decrease in maximum load but a significant decrease in the modulus of elasticity as well. The Wilcoxon test further confirmed significant differences before and after heat treatment for both materials. These findings, while preliminary due to the pilot nature of the study, emphasize the substantial impact of heat treatment on mini-implant mechanical properties and highlight the necessity for optimized protocols in clinical applications.

Processing parameters and heat treatment have a significant impact on the microstructure and porosity of Ti6Al4V and 316L alloys, thereby affecting their biocompatibility and integration as orthodontic mini-implants. Research has demonstrated that microstructural characteristics, such as grain size and phase fractions, are crucial in the machining of heat-treated Ti6Al4V titanium alloys [15]. The Ti6Al4V alloy and 316L exhibit differences in hardness, elastic modulus, and tensile strength. The Ti6Al4V alloy generally has higher hardness, elastic modulus, and tensile strength compared to 316L stainless steel [16]. The Ti6Al4V alloy is known for its higher strength, lower modulus of elasticity, better corrosion resistance, and superior biocompatibility, making it a preferred material for orthodontic mini-implants [17]. Especially 316L stainless steel is widely utilized as a material in various applications due to its superior corrosion resistance and good mechanical properties [18]. The hardness and elastic modulus of Ti6Al4V alloys are influenced by factors such as the orientation of the α and β phases within the alloy [19]. However, Ti alloys, including Ti6Al4V, are noted for their high notch sensitivity, which can affect fatigue resistance due to the presence of defects that act as stress raisers [20]. In orthodontic applications, the choice of material for mini-implants is crucial. The use of Ti6Al4V alloys for mini-implants is common due to their mechanical properties, biocompatibility, and strength, which are essential for providing stable anchorage during orthodontic treatment [21]. The mismatch in Young’s modulus values between the bone and the implant, as seen with materials like 316L stainless steel, can lead to stress-shielding effects and potential implant loosening [22]. Overall, the differences in hardness, elastic modulus, and tensile strength between Ti6Al4V alloys and 316L have significant implications for the behavior and performance of orthodontic mini-implants. The superior mechanical properties and biocompatibility of Ti6Al4V make it a preferred choice for ensuring the stability and success of orthodontic treatments involving mini-implants. The mechanical properties of Ti6Al4V alloys and 316L are crucial for their tribological behavior, wear resistance, and corrosion resistance, which are essential factors for their biocompatibility and integration into adjacent tissues. Ti6Al4V alloys are widely used in orthopedic implants due to their adequate mechanical properties, corrosion resistance, and biocompatibility [23]. Additionally, Ti6Al4V has excellent mechanical properties, corrosion resistance, and superplasticity, making it a favorable material for various applications [24].

The results obtained indicate that the increase in maximum displacement suggests greater deformation under the same load, implying a change in material behavior during heat treatment. The decrease in elastic modulus indicates lower resistance to elastic deformation, possibly due to microstructural changes induced by heat treatment. Reduced stiffness indicates the lower resistance of the material to deformation. The increase in hardness may suggest better resistance to plastic deformation, which could be advantageous in specific applications. A similarity between the elastic modulus of the implant material and that of human bones can mitigate the risk of bone resorption and enhance implant integration [25,26,27]. Human bones typically have an elastic modulus ranging from 10 to 30 GPa, making both Ti6Al4V and 316L suitable choices in this aspect. Having an elastic modulus closer to that of natural bones facilitates a more even distribution of forces during chewing, thereby lowering the risk of implant or adjacent bone damage. It is crucial to interpret these findings from the provided tables to comprehend how heat treatment has impacted the properties of the investigated materials, specifically the Ti6Al4V alloy and the 316L stainless steel.

The results gleaned from the indentation method for the examined alloys have revealed a marginal variance in elastic modulus. Specifically, for Ti6Al4V and 316L, the elastic modulus exhibited a decrease compared to the standard value. Guo et al. emphasized that the elastic modulus of Ti6Al4V significantly surpasses that of human cortical bone [28]. Similarly, the elastic modulus of stainless steel 316L greatly exceeds that of human bones, rendering implants prone to failure due to the stress-shielding phenomenon [29]. This disparity in elastic moduli can instigate stress-shielding effects, culminating in bone resorption and implant failure [30]. Additionally, Piotrowski et al. [31] underscored the elastic modulus’s impact on load transfer between the implant and bone during loading, indicating that the implant material’s elastic modulus wields a significant influence on bone response and stress distribution. Moreover, Zhang et al. [32] demonstrated that implants with lower elastic moduli could enhance bone growth volume by optimizing force transmission, signifying a favorable impact of lower elastic moduli on bone response and osseointegration. Collectively, these studies underscore the finding that the elastic modulus of implant materials profoundly influences bone’s response to mini-implants in terms of remodeling, osseointegration, and bone resorption.

The design and material properties of the implant, including its elastic modulus, play a pivotal role in determining biomechanical interaction with surrounding bone tissue. The post-heat treatment elastic modulus results obtained via the indentation method indicate that for Ti6Al4V, it was 76.81 GPa, and for 316L, it was 157.87 GPa. Heat treatment applied to Ti6Al4V alloy mini-implants reduced the elastic modulus by roughly 26.14%, and for 316L, the decrease was approximately 24.82%. These findings denote alterations in the mechanical properties of the materials due to the applied heat treatment. Understanding the effects of heat treatments on the material properties of Ti6Al4V and 316L alloys is imperative for comprehending how these alloys perform in orthodontic settings with mini-implants. Heat treatments can induce significant changes in the microstructure and mechanical properties of these alloys, thereby influencing their potential applications in orthodontics. In the case of Ti6Al4V, parameters such as temperature, cooling method, and exposure duration have been identified as factors influencing microstructure and hardness properties [33].

Post-heat treatment analyses on Ti6Al4V have unveiled significant changes in grain size and mechanical properties of the material [34]. Similarly, for stainless steel 316L, it has been observed that heat treatments impact mechanical properties, fatigue behavior, and corrosion resistance, all crucial aspects in the realm of orthodontic mini-implants [35]. The changes induced by heat treatments can directly impact the performance of these alloys in orthodontics with mini-implants. For instance, the fatigue behavior of stainless steel 316L is influenced by stress relaxation heat treatment, vital for ensuring the reliability of mini-implants during orthodontic procedures [36]. Additionally, aspects such as the microstructure and porosity of alloys, influenced by processing parameters and heat treatment, represent significant considerations for biocompatibility and tissue integration. Research has shown that the evolution of the melt zone, porosity, and microstructures in Ti6Al4V parts fabricated through selective laser melting can be influenced by processing parameters, thus impacting the biocompatibility and integration of these alloys into adjacent tissues [37].

Regarding applicability in orthodontic practice, the findings suggest that heat treatment can significantly impact the mechanical properties of orthodontic mini-implants made from Ti6Al4V and 316L alloys. Understanding these changes is crucial for orthodontic practitioners when selecting and utilizing these materials in clinical settings. Specifically, knowledge of how heat treatment affects properties such as deformation, elasticity, stiffness, and hardness can guide clinicians in determining the optimal mini-implant material and treatment approach for individual patients.

However, it is important to note some limitations of the study. Firstly, the investigation focused on mechanical properties only, and other factors such as biocompatibility, corrosion resistance, and long-term clinical performance were not addressed. Additionally, the study was conducted under controlled laboratory conditions, and the findings may not fully represent the complex in vivo environment in orthodontic applications. Furthermore, the sample size and specific characteristics of the mini-implants tested may limit the generalizability of the results to all orthodontic mini-implants on the market.

## 5. Conclusions

The thermal influence on mini-implants can significantly affect their mechanical characteristics and their compatibility with surrounding bone tissue in terms of biomechanics. Heat treatment becomes crucial in the development and utilization of orthodontic mini-implants to attain optimal outcomes in orthodontic therapy. Nonetheless, further investigation is necessary to comprehensively grasp the implications of heat treatment on mini-implants and to refine the design and efficacy of these devices in clinical settings.

## Figures and Tables

**Table 1:** Mean values of indentation test results for the investigated samples before and after heat treatment.

Sample	Maximum Load Before [n]	Maximum Load After [n]	Maximum Displacement Before [µm]	Maximum Displacement After [µm]	Maximum Displacement Difference [µm]	Modulus of Elasticity Before [gpa]	Modulus of Elasticity After [gpa]	Modulus of Elasticity Difference [gpa]	Stiffness Before [n/µm]	Stiffness After [n/µm]	Stiffness Difference [n/µm]	Hardness Before [gpa]	Hardness After [gpa]	Hardness Difference [gpa]
Group 1 TI6AL4V alloy	4.50	4.50	8.22	9.21	0.99	105	76.81	−28.19	1.554	0.91	−0.644	2.06	2.45	0.39
Group 2 316L	4.50	4.50	6.62	7.54	0.92	210	157.87	−52.13	2.074	1.93	−0.144	0.87	0.63	−0.24

## Data Availability

Data are contained within the article.

## References

[1] Chandak A.M., Tarvade S.D., Sharma M., Kaurani H.J. (2023). In-vitro investigation of primary stability of orthodontic mini implants with different lengths using resonance frequency analysis. J. Contemp. Orthod..

[2] Razaghi P., Haghgou J.M., Khazaei S., Farhadian N., Fekrazad R., Gholami L. (2022). The effect of photobiomodulation therapy on the stability of orthodontic mini-implants in human and animal studies: A systematic review and meta-analysis. J. Lasers Med. Sci..

[3] Hilloulin B., Lagrange M., Duvillard M., Garioud G. (2022). ε-greedy automated indentation of cementitious materials for phase mechanical properties determination. Cem. Concr. Compos..

[4] Anbarasu P., Ramesh B., Annamalai I., Subramanian S. (2022). Mini implant ‘safe zones’ in orthodontics: A comprehensive review. Arch. Dent. Res..

[5] Migliorati M., Drago S., Amorfini L., Nucera R., Silvestrini-Biavati A. (2020). Maximum insertion torque loss after miniscrew placement in orthodontic patients: A randomized controlled trial. Orthod. Craniofacial Res..

[6] Wilmes B., Panayotidis A., Drescher D. (2011). Fracture resistance of orthodontic mini-implants: A biomechanical in vitro study. Eur. J. Orthod..

[7] Herrera S., Diez-Perez A. (2017). Clinical experience with microindentation in vivo in humans. Bone.

[8] Zhang Q., Le Roy R., Vandamme M., Zuber B. (2014). Long-term creep properties of cementitious materials: Comparing microindentation testing with macroscopic uniaxial compressive testing. Cem. Concr. Res..

[9] Arnold M., Zhao S., Ma S., Giuliani F., Hansen U., Cobb J.P., Abel R.L., Boughton O. (2017). Microindentation—A tool for measuring cortical bone stiffness?. Bone Jt. Res..

[10] Ryu J.-H., Kwon J.-S., Jiang H.B., Cha J.-Y., Kim K.-M. (2018). Effects of thermoforming on the physical and mechanical properties of thermoplastic materials for transparent orthodontic aligners. Korean J. Orthod..

[11] Zysset P.K. (2009). Indentation of bone tissue: A short review. Osteoporos. Int..

[12] Parthasarathy J., Starly B., Raman S., Christensen A. (2010). Mechanical evaluation of porous titanium (Ti6Al4V) structures with electron beam melting (EBM). J. Mech. Behav. Biomed. Mater..

[13] Frost H.M. (2004). A 2003 update of bone physiology and wolff’s law for clinicians. Angle Orthod..

[14] He R., Gahlawat S., Guo C., Chen S., Dahal T., Zhang H., Liu W., Zhang Q., Chere E., White K. (2015). Studies on mechanical properties of thermoelectric materials by nanoindentation. Phys. Status Solidi A.

[15] Ahmadi M., Karpat Y., Acar O., Kalay Y.E. (2018). Microstructure effects on process outputs in micro scale milling of heat treated Ti6Al4V titanium alloys. J. Am. Acad. Dermatol..

[16] Hasiak M., Sobieszczańska B., Łaszcz A., Biały M., Chęcmanowski J., Zatoński T., Bożemska E., Wawrzyńska M. (2021). Production, mechanical properties and biomedical characterization of zrti-based bulk metallic glasses in comparison with 316L stainless steel and Ti6Al4V Alloy. Materials.

[17] Mierzejewska Ż.A., Hudák R., Sidun J. (2019). Mechanical properties and microstructure of dmls TI6AL4V alloy dedicated to biomedical applications. Materials.

[18] Li D., Wang J., Chen D., Liang P. (2015). Influence of molybdenum on tribo-corrosion behavior of 316l stainless steel in artificial saliva. J. Bio- Tribo-Corros..

[19] He Z., He H., Lou J., Li Y., Li D., Chen Y., Liu S. (2020). Fabrication, structure and mechanical and ultrasonic properties of medical TI6AL4V alloys part i: Microstructure and mechanical properties of TI6AL4V alloys suitable for ultrasonic scalpel. Materials.

[20] Dallago M., Fontanari V., Torresani E., Leoni M., Pederzolli C., Potrich C., Benedetti M. (2018). Fatigue and biological properties of Ti-6Al-4V ELI cellular structures with variously arranged cubic cells made by selective laser melting. J. Mech. Behav. Biomed. Mater..

[21] Redžepagić-Vražalica L., Mešić E., Pervan N., Hadžiabdić V., Delić M., Glušac M. (2021). Impact of implant design and bone properties on the primary stability of orthodontic mini-implants. Appl. Sci..

[22] Prasadh S., Ratheesh V., Manakari V., Parande G., Gupta M., Wong R. (2019). The potential of magnesium based materials in mandibular reconstruction. Metals.

[23] Anaya-Garza K., Domínguez-Crespo M.A., Torres-Huerta A., Palma-Ramírez D., Rodríguez-Salazar A.E., Ramírez-Meneses E., Brachetti-Sibaja B. (2021). Electrochemical performance of uhmwpe biocompatible coatings on Ti6Al4V alloy in simulated body solutions: Influence of immersion time. ECS Trans..

[24] Taran V., Garkusha I., Taran A., Muratov R., Vorontsov P., Gnidenko Y., Herasimov H., Starikov V., Baturin A., Romaniuk S. (2020). Functional protective zrn coatings on implants for trauma surgery. Probl. At. Sci. Technol..

[25] Baltatu M.S., Spataru M.C., Verestiuc L., Balan V., Solcan C., Sandu A.V., Geanta V., Voiculescu I., Vizureanu P. (2021). Design, synthesis, and preliminary evaluation for Ti-Mo-Zr-Ta-Si alloys for potential implant applications. Materials.

[26] Baltatu I., Sandu A.V., Vlad M.D., Spataru M.C., Vizureanu P., Baltatu M.S. (2022). Mechanical characterization and in vitro assay of biocompatible titanium alloys. Micromachines.

[27] Baltatu M.S., Vizureanu P., Sandu A.V., Florido-Suarez N., Saceleanu M.V., Mirza-Rosca J.C. (2021). New titanium alloys, promising materials for medical devices. Materials.

[28] Guo Y., Chen D., Cheng M., Lu W., Wang L., Zhang X. (2013). The bone tissue compatibility of a new Ti35Nb2Ta3Zr alloy with a low young’s modulus. Int. J. Mol. Med..

[29] Essa K., Jamshidi P., Zou J., Attallah M.M., Hassanin H. (2018). Porosity control in 316L stainless steel using cold and hot isostatic pressing. Mater. Des..

[30] Raganya L., Moshokoa N., Machaka R., Obadele B., Makhatha M. (2022). Microstructure and tensile properties of heat-treated Ti-Mo alloys. MATEC Web Conf..

[31] Piotrowski B., Baptista A., Patoor E., Bravetti P., Eberhardt A., Laheurte P. (2014). Interaction of bone–dental implant with new ultra low modulus alloy using a numerical approach. Mater. Sci. Eng. C.

[32] Zhang Y., Wang J., Wang P., Fan X., Li X., Fu J., Guo Z. (2012). Low elastic modulus contributes to the osteointegration of ti-tanium alloy plug. J. Biomed. Mater. Res. Part B Appl. Biomater..

[33] Lekoadi P., Tlotleng M., Annan K., Maledi N., Masina B. (2021). Evaluation of heat treatment parameters on microstructure and hardness properties of high-speed selective laser melted Ti6Al4V. Metals.

[34] Jebieshia T.R., Kim J.M., Kang J.W., Son S.W., Kim H.D. (2020). Microstructural and very high cycle fatigue (vhcf) behavior of Ti6Al4V—A comparative study. Materials.

[35] Lei J., Ge Y., Liu T., Wei Z. (2021). Effects of heat treatment on the microstructure and mechanical properties of selective laser melting 316L stainless steel. Shock. Vib..

[36] Elangeswaran C., Cutolo A., Muralidharan G.K., de Formanoir C., Berto F., Vanmeensel K., Van Hooreweder B. (2019). Effect of post-treatments on the fatigue behaviour of 316L stainless steel manufactured by laser powder bed fusion. Int. J. Fatigue.

[37] Peng H., Fang W., Dong C., Yi Y., Wei X., Luo B., Huang S. (2021). Nano-mechanical properties and creep behavior of TI6AL4V fabricated by powder bed fusion electron beam additive manufacturing. Materials.

